# Supplementary material for: Time-of-day effects of cancer drugs revealed by high-throughput deep phenotyping
Source: Nat Commun. 2024 Aug 22;15:7205. doi: 10.1038/s41467-024-51611-3 (PMC11339390; doi:10.1038/s41467-024-51611-3)
Supplement: Supplementary file 1 — Supplementary Information [file 41467_2024_51611_MOESM1_ESM.pdf]

Supplementary Information

for

Time-of-day effects of cancer drugs revealed by high-throughput deep phenotyping

Carolin Ector, Christoph Schmal, Jeff Didier, Sébastien De Landtsheer, Anna-Marie Finger, Francesca Müller-Marquardt, Johannes H. Schulte, Thomas Sauter, Ulrich Keilholz, Hanspeter Herzel, Achim Kramer, Adrián E. Granada

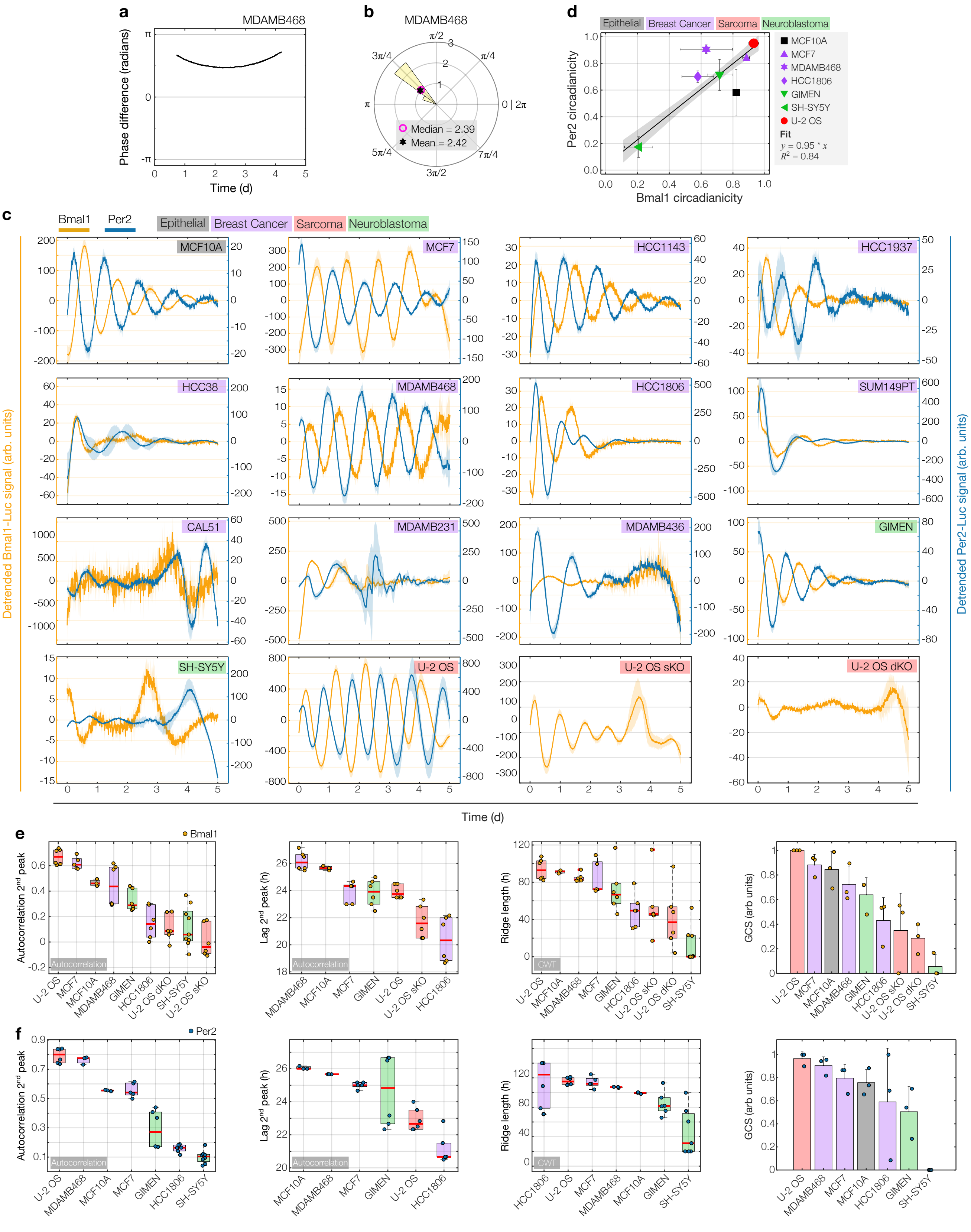

Supplementary Fig. 1 | Complementary analysis to deep circadian phenotyping approach.

a, Example of absolute phase difference evolution between *Bmal1*- and *Per2*-Luc signals, here shown for single samples of MDAMB468 cells. **b,** Polar histogram depicting the phase difference between *Bmal1* and *Per2* signals in MDAMB468 cells over time. 2π denotes one full circadian cycle. Median

and mean phase difference are indicated as pink circle and black star, respectively. **c,** Detrended *Bmal1*-Luc (yellow) and *Per2*-Luc (blue) signals of all cell lines used in this study. Data represents the mean±s.d. of 3 technical replicates recorded on the same day. **d,** Linear regression analysis of the MRA circadian component ('circadianicity') of *Bmal1*- versus *Per2*-Luc signals. Markers indicate different cell models. Grey-shaded area=95% CI. Model accuracy indicated by R^2-values. Data represents the mean±s.d with *n*=6 samples for each reporter, collected from biological duplicates in technical triplicates or duplicates (HCC1806 *Per2*-Luc). For MCF10A-*Bmal1*-Luc, MCF10A-*Per2*-Luc and MDAMB468-*Per2*-Luc: *n*=3 technical triplicates measured in a single experiment. *n*=9 or 8 for SH-SY5Y-*Bmal1*-Luc or *Per2*-Luc, respectively, collected from biological triplicates with technical triplicates or duplicates. **e** and **f,** *Bmal1*-dependent (**e**) and *Per2*-dependent (**f**) circadian analysis corresponding to the rankings of the combined data shown Fig. 2e, f, h, and k of the main article. Box bounds are defined by the 25th and 75th percentiles. Extending whiskers represent data points within 1.5 times the interquartile range from lower and upper quartiles. Red lines denote the data's median. For sample size see (**d**). Color-coding of cell models in **c-f** according to their tissue of origin. Source data are provided as a Source Data file.

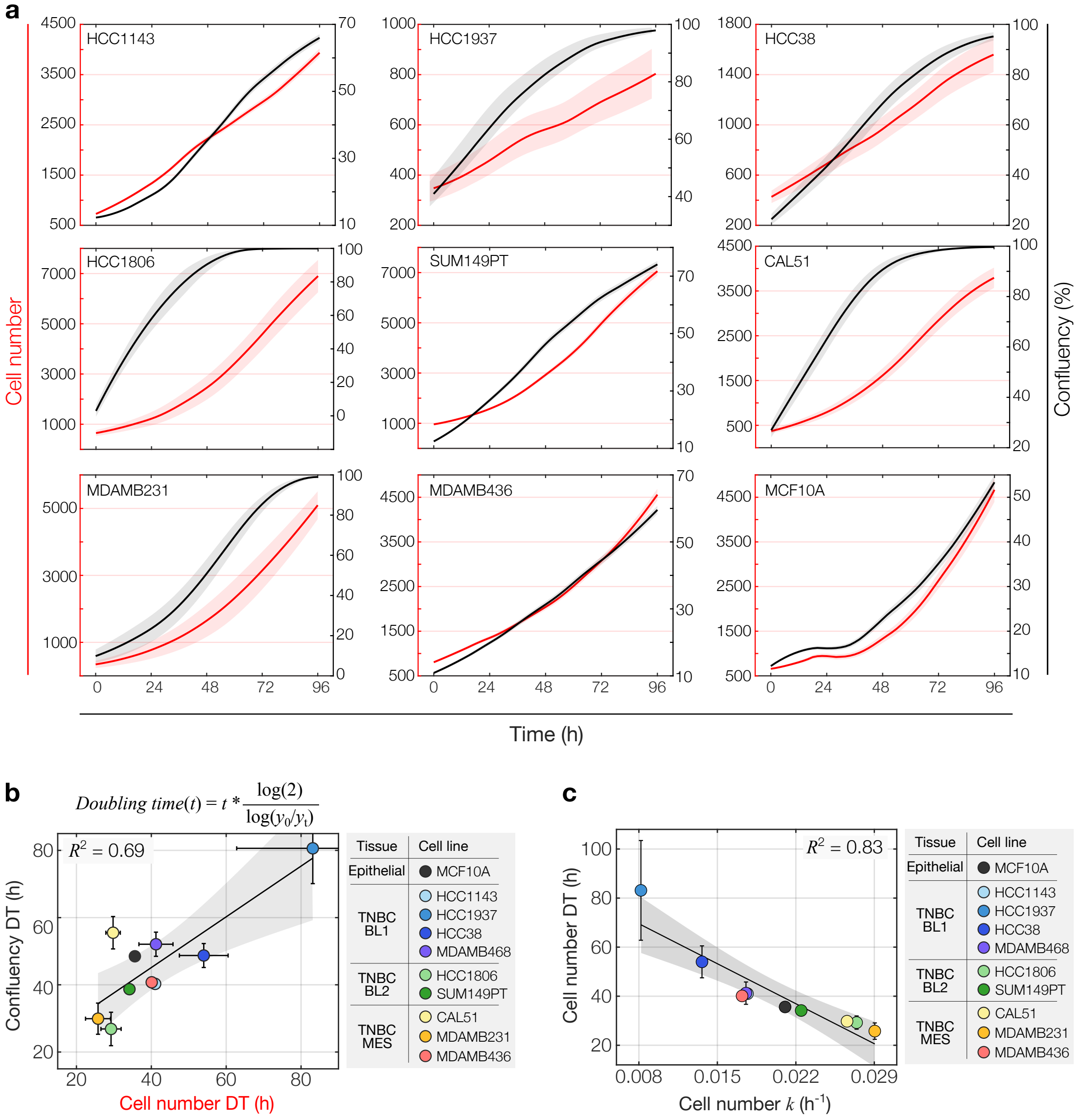

Supplementary Fig. 2 | Complementary analysis to characterization of growth dynamics.

a, Growth curves of the indicated cell lines monitored by live-cell imaging for 4 days and quantified by either cell numbers (red line) or confluency (black line). Data represents the mean±s.e.m. of 9 images taken per cell line on a single plate. **b,** Linear regression analysis of doubling times (DT's) calculated from confluency versus cell numbers. **c,** Linear regression analysis of the doubling time and growth rate (*k*) calculated from cell numbers of various cell lines. Color coding of cell models in (**b**) and (**c**) according to their tissue of origin. Data shown in (**b**) and (**c**) represents the mean±s.d. (DT's, across 9 images taken per cell line on a single plate), or ±CI of the exponential fit (*k*-values). Grey-shaded area=95% CI. Model accuracy indicated by R^2-values. Source data are provided as a Source Data file.

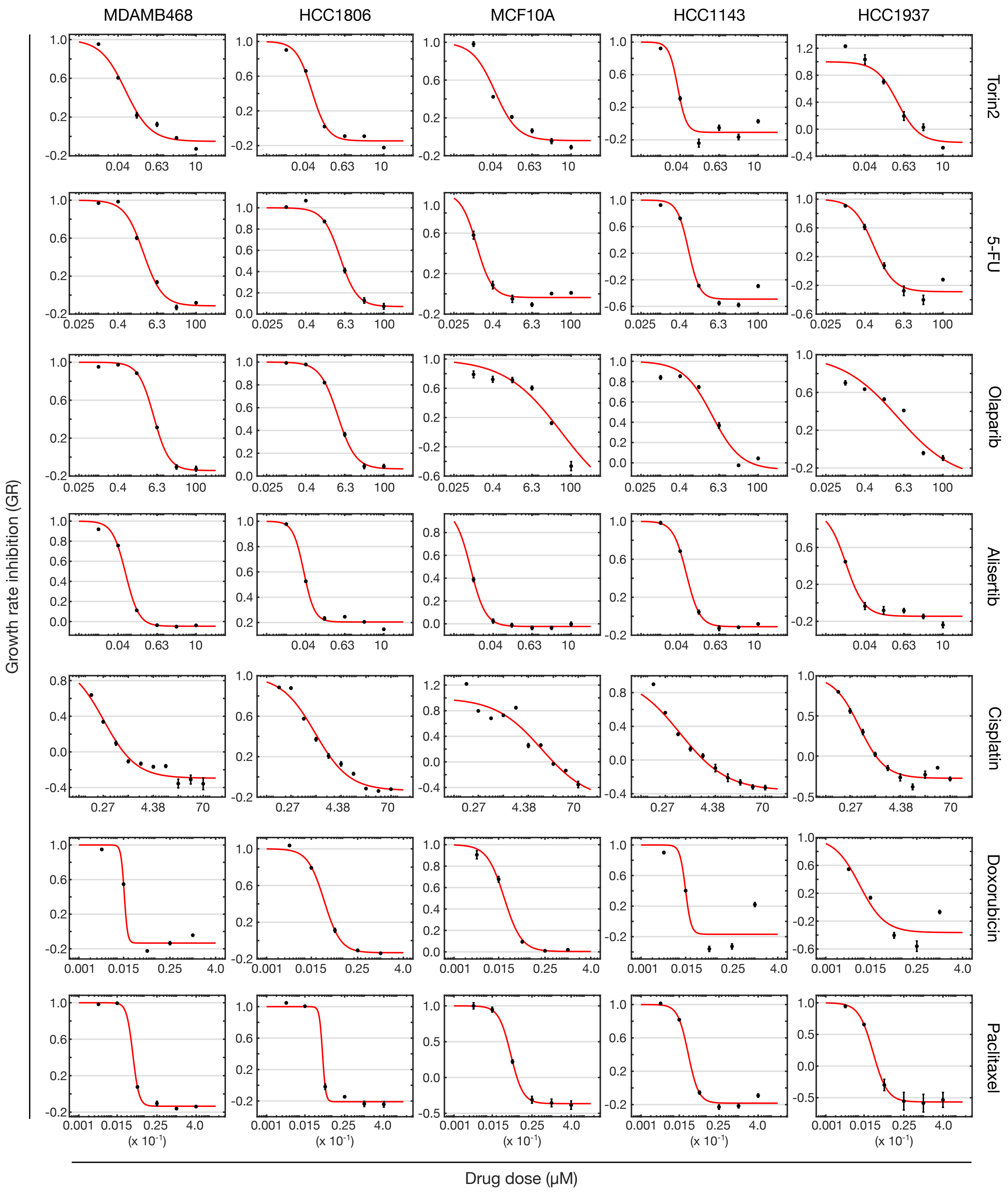

Supplementary Fig. 3 | Dose-response curves of *GR*-values for multiple cell models and drugs. The data is based on the mean responses of technical duplicates, live-imaged on independent assay plates for 4 days. For cisplatin, a single sample was imaged with 9 images taken per condition. Error bars indicate the 95% CI of the exponential fit. Source data are provided as a Source Data file.

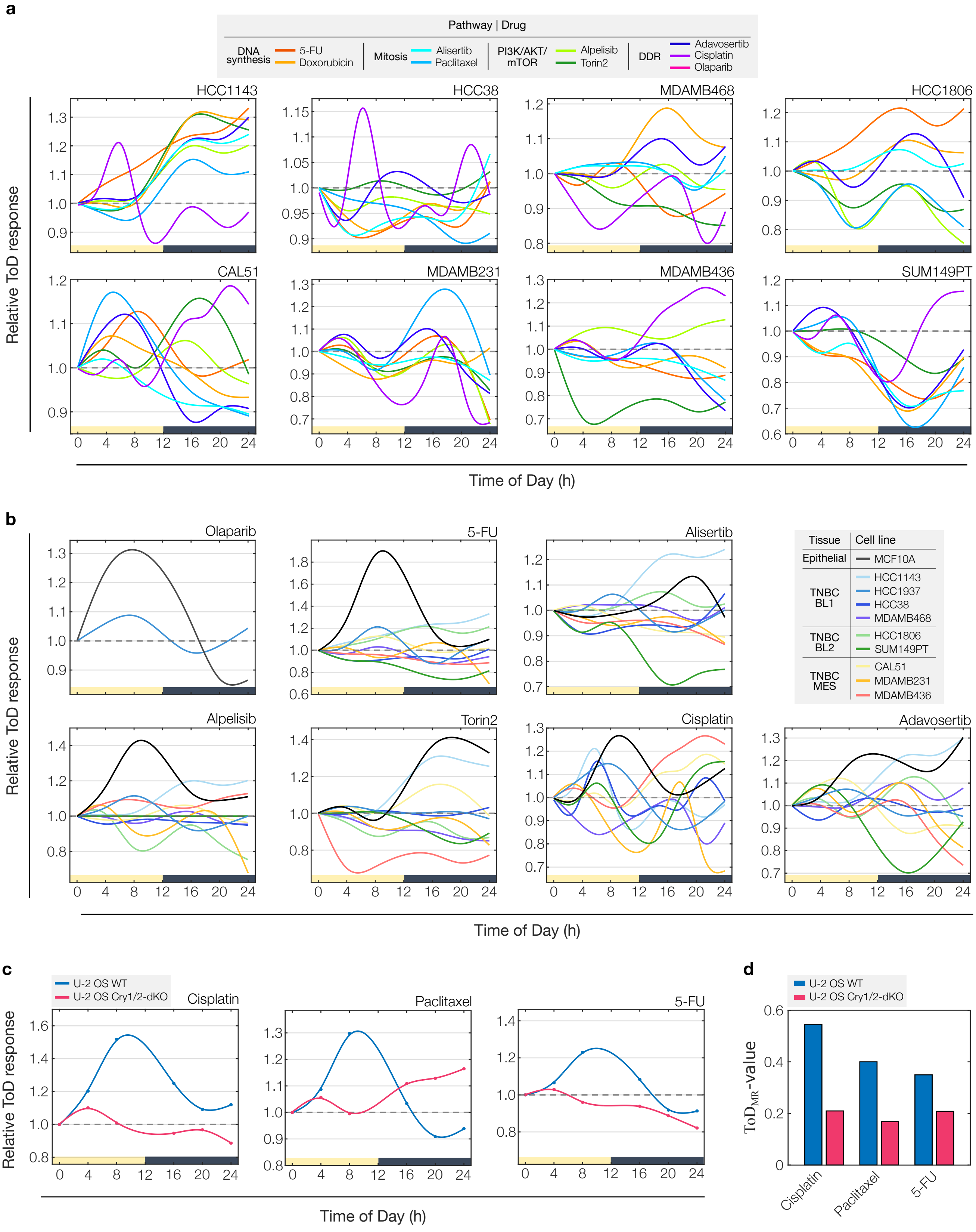

Supplementary Fig. 4 | Complementary analysis to time-of-day sensitivity characterizations.

a, Overlay of ToD response curves (ToD-RCs) for individual cell models treated with different drugs (color-coded). **b,** Overlay of ToD-RCs for multiple cell models (color-coded), grouped by the administered drug. Data in (**a**) and (**b**) represents the mean for each drug-cell combination tested on

two plates (n=2 technical replicates) or across 9 images taken per condition (cisplatin). **c,** Overlay of ToD-RCs for U-2 OS wildtype and circadian *Cry1*/*Cry2*-double knock-out cell lines, grouped by the administered drugs. Cell line models are color-coded. Data represents the mean of two plates (n=2 technical replicates). **d,** Ranking of ToD_{MR}-values for each drug-cell combination of the ToD-RCs shown in (**c**). Source data are provided as a Source Data file.

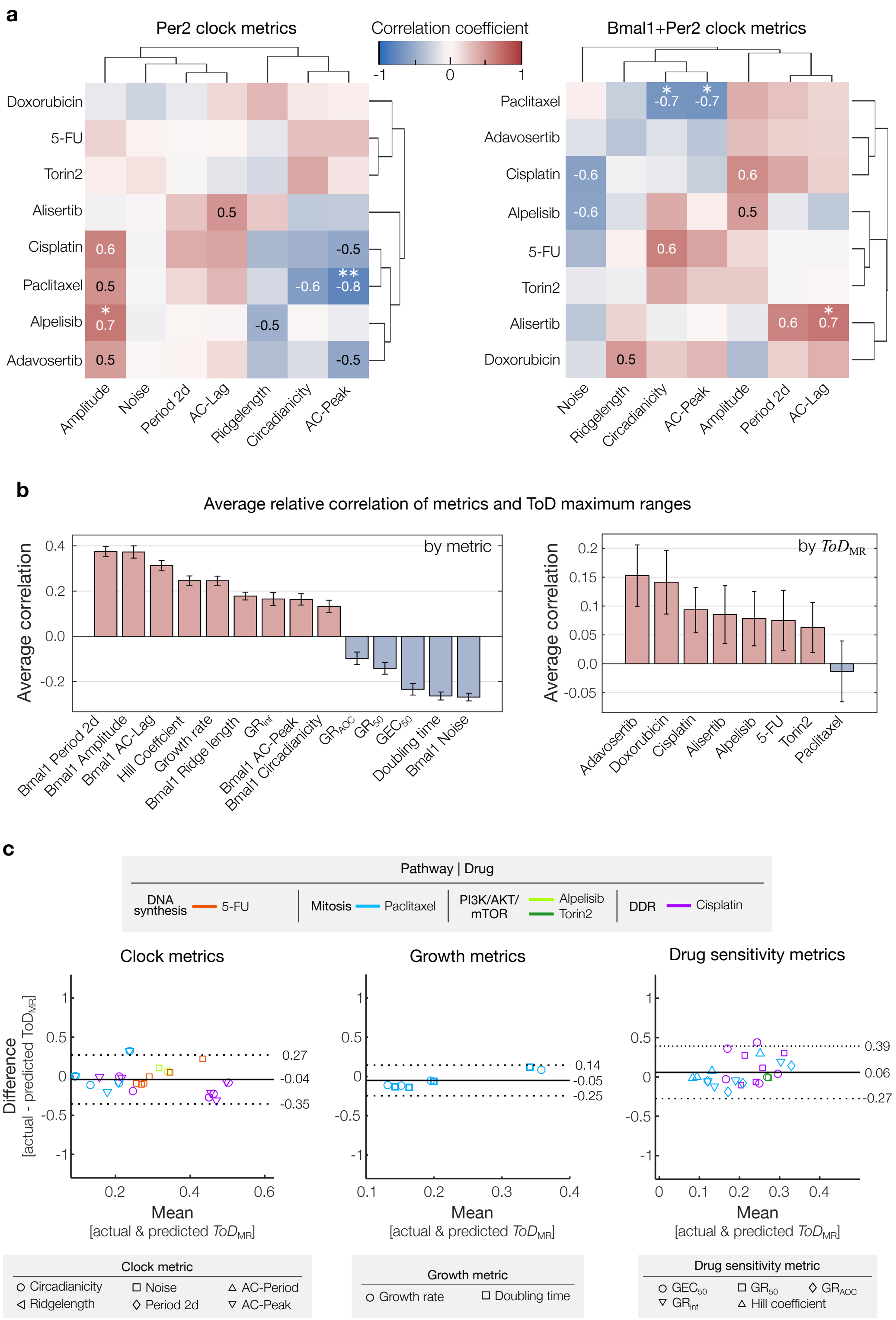

Supplementary Fig. 5 | Complementary analysis to cellular determinants of time-of-day sensitivity.

a, Hierarchical clustering of correlation coefficients between ToD_{MR}-values for different drugs (rows) and clock strength metrics (columns) obtained from the *Per2*-reporter (left) or calculated as a mean from both, *Bmal1*- and *Per2*-reporters (right). Significant pairwise correlations (two-sided test with no adjustments made) are indicated by stars, where * and **, denote *p*-values ≤ 0.05 and 0.01,

respectively. Exact *p*-values: Paclitaxel/*Per2*-AC-Peak=0.007; Alpelisib/*Per2*-Amplitude=0.049; Paclitaxel/*Mixed*-AC-Peak=0.028; Paclitaxel/*Mixed*-Circadianicity=0.031; Alisertib/*Mixed*-AC-Lag=0.032. *n*=9 (cisplatin and alpelisib) or *n*=10 cell models (all other drugs). **b,** Bar plots ranking relative overall correlation between ToD_{MR}-values and individual cellular metrics (depicted in Fig. 5d–f of the main article), averaged by metric (left, *n*=8 drugs) or by drug (right, *n*=14 metrics). Positive and negative correlation values are color-coded in light red and blue, respectively. Data represents the mean±s.e.m. **c,** Bland-Altman plots comparing predicted and actual ToD_{MR}-values of new data that was not used to derive the original statistical associations shown in Fig 5d-f of the main article, including new datapoints for the clock (*n*=26), growth (*n*=8), and drug sensivity (*n*=24) datasets, respectively (refer to Supplementary Data 3 for full new datatset). The predictions were based on fitting a linear regression model to the original data associated with Pearson correlation coefficients ≥ 0.5. The individual parameters considered for each dataset are distinguished by marker, different drugs are uniformly color-coded across all three datasets. The central solid line indicates the mean bias between the predicted and actual ToD_{MR}-values of the new data and the outer lines mark the upper and lowers limits of agreement (±1.96*s.d.). Source data are provided as a Source Data file.

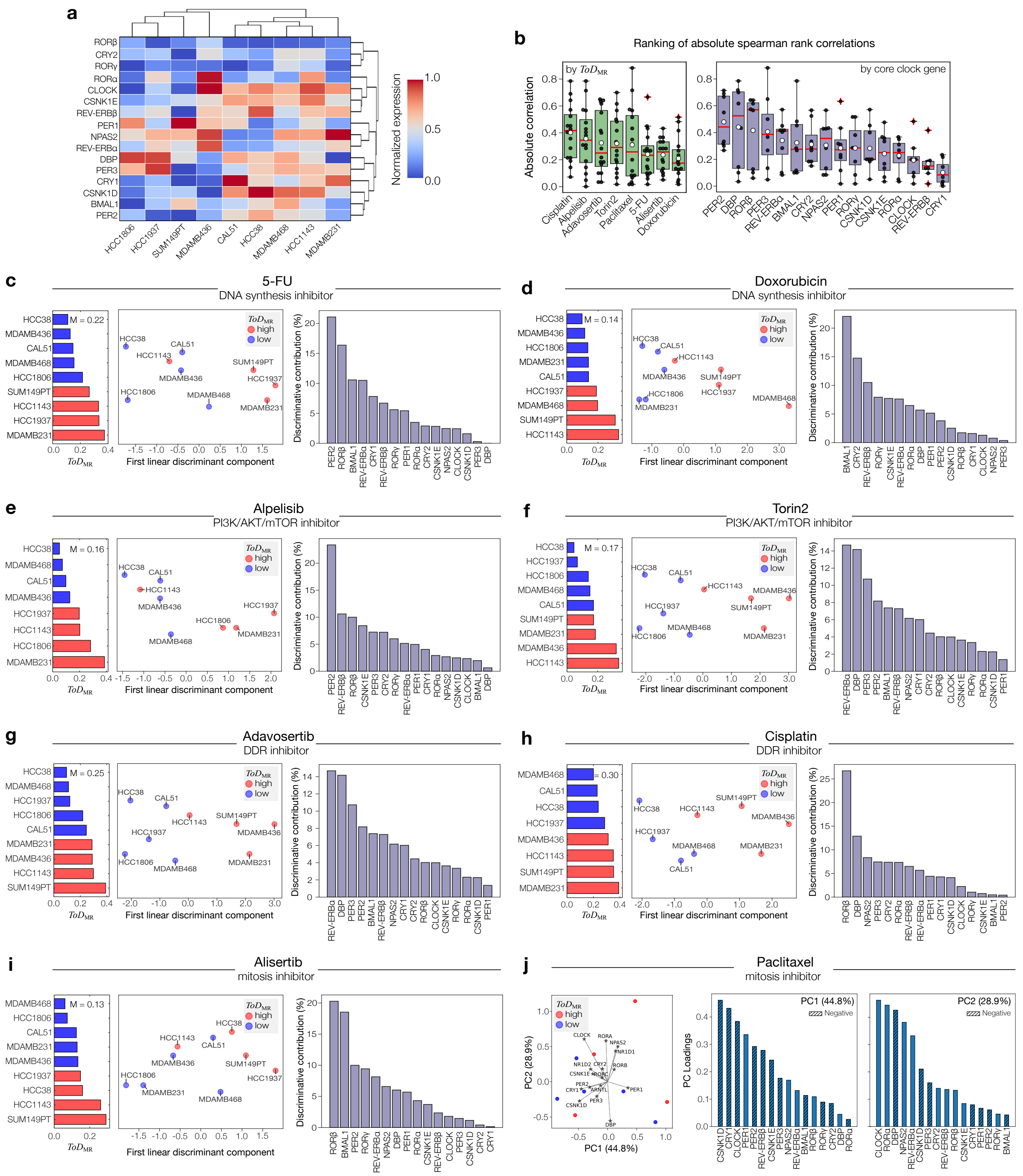

Supplementary Fig. 6 | Complementary analysis to the impact of core clock genes in shaping time-of-day sensitivity.

a, Gene expression clustermap of the selected list of core clock genes (rows) for the different cell models (columns) used in this work. **b,** Boxplot showing the mean-based ranking of absolute spearman rank correlation values between circadian clock gene expression levels and ToDMR values, categorized by drug (*n*=16 circadian clock genes) or by circadian clock gene (*n*=8 drugs). Bottom and top edges of the boxes indicate the 25th and 75th percentiles, respectively. Red horizontal lines denote the median, white circles the mean, and red crosses indicate outliers. **c-i,** Linear discriminant analysis

on median-based binarized ToD_{MR}-values for the indicated drugs. ToD_{MR}-values below or above the median (M) are color-coded as blue and red, respectively (middle and left panel of each subfigure). The contribution to the obtained discriminative information is shown in percentage for each of the circadian clock genes (right panel of each subfigure). **j,** Principal Component Analysis (PCA) biplot of the first two principal components on ToD sensitivity groups related to paclitaxel, with ToD sensitivity groups color-coded. Overlaid are the principal component scores for circadian clock genes, scaled to demonstrate their relative contribution to determining ToD sensitivity. The percentage of variance explained by each principal component is denoted by their respective loadings (left panel). The bar charts detail the ranked contribution of individual circadian clock genes to PC1 and PC2, presented in descending order (middle and right panels, respectively). Source data are provided as a Source Data file.

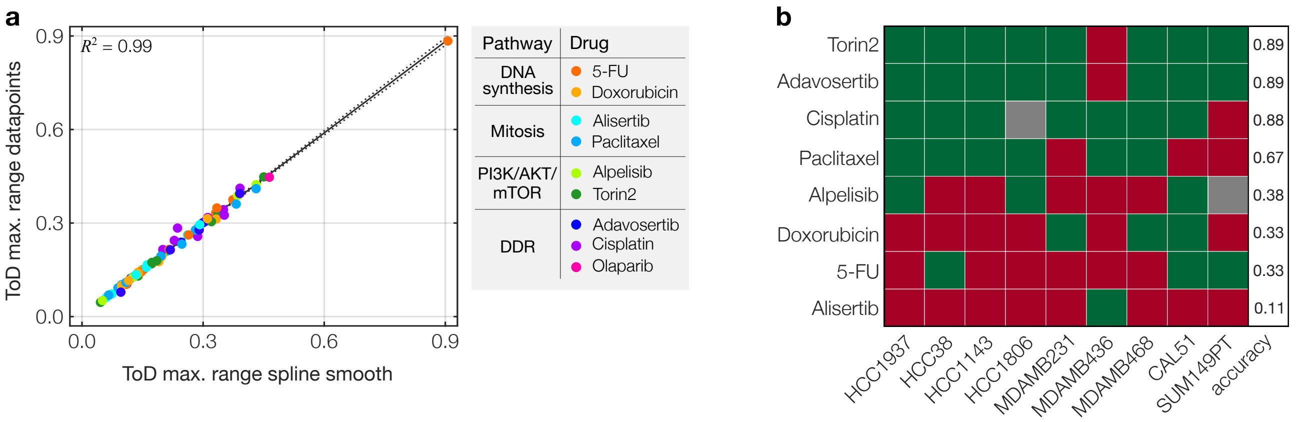

Supplementary Fig. 7 | Evaluation of methodological approaches.

a, Linear regression analysis of the maximum range in ToD responses (ToD_{MR}), determined from the discrete measured data points or from continuous spline smoothed ToD response curves. Drugs are color-coded. Dotted lines=95% CI. Model accuracy indicated by R^2-values. n=80 drug-cell combinations. **b,** Leave-one-out cross-validation test (LOOCV) on LDA results shown in Fig. 6d of the main article and Supplementary Fig. 6d-h, where the potential of circadian gene expression levels to discriminate between high and low ToD sensitivity groups (based on median-binarized ToD_{MR}-values) is investigated. The color coding reflects the accuracy of each cell line model in being correctly assigned to its ToD-sensitivity group, where green and red indicate true and false categorization, respectively. The rightmost column denotes the summed LOOCV accuracy per drug, where 1 refers to 100% accuracy. n=8 (cisplatin and Alpelisib) or 9 cell line models (all other drugs). Source data are provided as a Source Data file.
