# Supplementary material for: Time-of-day effects of cancer drugs revealed by high-throughput deep phenotyping
Source: Nat Commun. 2024 Aug 22;15:7205. doi: 10.1038/s41467-024-51611-3 (PMC11339390; doi:10.1038/s41467-024-51611-3)
Supplement: Supplementary file 2 — Description of Additional Supplementary Files [file 41467_2024_51611_MOESM2_ESM.pdf]

Description of Supplementary Data

Supplementary Data 1. Tables including drug sensitivity parameters and/or used doses for the data underlying Figures 3j–m. Drug sensitivity parameters of all cell model – drug combinations utilized for calculating the Pearson's correlation coefficients shown in Figure 3n and for correlation and importance analysis shown in Figure 5c, f-i.

Supplementary Data 2. Table including *Bmal1*, *Per2* and *Bmal1-Per2* (mixed) circadian metrics of all cell models analyzed in Figure 5.

Supplementary Data 3. New dataset including *Bmal1* circadian, growth, drug sensitivity and ToD_{MR} metrics of up to six cell line models used for the Blans-Altman plots shown in Supplementary Figure 5c.
