# Supplementary material for: Time-of-day effects of cancer drugs revealed by high-throughput deep phenotyping
Source: Nat Commun. 2024 Aug 22;15:7205. doi: 10.1038/s41467-024-51611-3 (PMC11339390; doi:10.1038/s41467-024-51611-3)
Supplement: Supplementary file 3 — Reporting Summary [file 41467_2024_51611_MOESM3_ESM.pdf]

nature portfolio

Corresponding author(s): Adrián E. Granada
Last updated by author(s): Jun 18, 2024

Reporting Summary

Nature Portfolio wishes to improve the reproducibility of the work that we publish. This form provides structure for consistency and transparency in reporting. For further information on Nature Portfolio policies, see our Editorial Policies and the Editorial Policy Checklist.

Statistics

For all statistical analyses, confirm that the following items are present in the figure legend, table legend, main text, or Methods section.

n/a	Confirmed	
[ ]	[x]	The exact sample size (*n*) for each experimental group/condition, given as a discrete number and unit of measurement
[ ]	[x]	A statement on whether measurements were taken from distinct samples or whether the same sample was measured repeatedly
[ ]	[x]	The statistical test(s) used AND whether they are one- or two-sided<br>*Only common tests should be described solely by name; describe more complex techniques in the Methods section.*
[x]	[ ]	A description of all covariates tested
[ ]	[x]	A description of any assumptions or corrections, such as tests of normality and adjustment for multiple comparisons
[ ]	[x]	A full description of the statistical parameters including central tendency (e.g. means) or other basic estimates (e.g. regression coefficient) AND variation (e.g. standard deviation) or associated estimates of uncertainty (e.g. confidence intervals)
[ ]	[x]	For null hypothesis testing, the test statistic (e.g. *F*, *t*, *r*) with confidence intervals, effect sizes, degrees of freedom and *P* value noted<br>*Give P values as exact values whenever suitable.*
[x]	[ ]	For Bayesian analysis, information on the choice of priors and Markov chain Monte Carlo settings
[x]	[ ]	For hierarchical and complex designs, identification of the appropriate level for tests and full reporting of outcomes
[ ]	[x]	Estimates of effect sizes (e.g. Cohen's *d*, Pearson's *r*), indicating how they were calculated

Our web collection on statistics for biologists contains articles on many of the points above.

Software and code

Policy information about availability of computer code

Data collection

Lumicycle analysis software (Actimetrics), for luciferase signal acquisition (https://actimetrics.com/products/lumicycle/).

Incucyte analysis software (Sartorius, v2022A), for acquisition and image analysis of live-cell imaging data.

Python programming language (v3.9.7) and PyCharm IDE (Community Edition 2021.2.2) for the import of gene expression data from the Cancer Cell Line Encyclopedia Dependency Map (CCLE DepMap).

The experimental raw data and data tables generated in this study have been deposited in the Figshare database under the identifier https://figshare.com/projects/Time-of-Day-Drug-Response/180916.

Data analysis

Data processing using the python language:
- Numpy v1.23.3 and pandas v1.4.4.

Bioluminescence time-series data:
- Anaconda Navigator v1.10.0: To operate the pyBOAT software.
- pyBOAT v0.9.1: For processing of bioluminescence data and continuous wavelet transform. Reference: Mönke, G. et al. Optimal time frequency analysis for biological data - pyBOAT. bioRxiv, 2020.2004.2029.067744 (2020).
- PyWavelets package v1.4.1 of the python language (v3.10.6): For multiresolution analysis. Reference: Lee, G. R. et al. PyWavelets: A Python package for wavelet analysis. Journal of Open Source Software 4. (2019).

Determinants of Time-of-Day sensitivity:
- SciPy v1.9.1 and uncertainties v3.1.7 packages of the python language (v3.10.6): For correlation analysis ('stats' and 'optimize' functions).
- dominance-analysis package of the python language: For Shapley value regression. Reference: https://dominance-analysis.github.io/dominance-analysis/

Drug sensitivity metrics:
- Calculations based on Hafner, M. et al. Growth rate inhibition metrics correct for confounders in measuring sensitivity to cancer drugs. Nature Methods 13, 521-527 (2016), implemented in MATLAB R2021b.

Gene expression data:
- seaborn library v0.11.2 of the python language (v. 3.9.7): For statistical visualization ('clustermap' and 'jointplot' functions).
- scikit-learn v1.1.1 of the python language (v. 3.9.7): sklearn discriminant_analysis module ('LinearDiscriminantAnalysis' function) and sklearn decomposition module ('PCA' function).

All other code for the analysis and visualization of time-series luciferase data or live-imaging growth data was implemented using MATLAB R2021b.

All code used for the data analysis in this work (in MATLAB and Python) is publicly available through the dataset repository Zenodo under the identifier https://zenodo.org/doi/10.5281/zenodo.11656060.

For manuscripts utilizing custom algorithms or software that are central to the research but not yet described in published literature, software must be made available to editors and reviewers. We strongly encourage code deposition in a community repository (e.g. GitHub). See the Nature Portfolio guidelines for submitting code & software for further information.

Data

Policy information about availability of data

All manuscripts must include a data availability statement. This statement should provide the following information, where applicable:

- Accession codes, unique identifiers, or web links for publicly available datasets
- A description of any restrictions on data availability
- For clinical datasets or third party data, please ensure that the statement adheres to our policy

Gene expression data of circadian clock genes were obtained from the Cancer Cell Line Encyclopedia Dependency Map (CCLE DepMap, https://sites.broadinstitute.org/ccle/datasets, Q4 of 2022). The experimental time series data and data tables for all results of this study are available on Figshare with the identifier https://figshare.com/projects/Time-of-Day-Drug-Response/180916.

Human research participants

Policy information about studies involving human research participants and Sex and Gender in Research.

Reporting on sex and gender	N/A
Population characteristics	N/A
Recruitment	N/A
Ethics oversight	N/A

Note that full information on the approval of the study protocol must also be provided in the manuscript.

Field-specific reporting

Please select the one below that is the best fit for your research. If you are not sure, read the appropriate sections before making your selection.

[x] Life sciences [ ] Behavioural & social sciences [ ] Ecological, evolutionary & environmental sciences

For a reference copy of the document with all sections, see nature.com/documents/nr-reporting-summary-flat.pdf

Life sciences study design

All studies must disclose on these points even when the disclosure is negative.

Sample size	Sample size was not pre-determined. According to the standards in the field, two independent bioluminescence experiments were conducted with two to three technical replicates. For live-cell imaging, we analyzed a minimum of four images for each condition, sourced from two independent plates.
Data exclusions	Data was retained for analysis except when identified as outliers through statistical testing, specifically using box plot methods.

Replication	Bioluminescence experiments were conducted twice to confirm their reproducibility. MCF10A Bmal1- and Per2-Luc and MDAMB468 Per2-Luc cells were not subjected to biological replication, though technical triplicates displayed high similiarity.<br><br>Live-imaging experiments were carried out a single time because of the comprehensive data generated. The evaluation of each condition was based on a detailed analysis of the captured individual images.
Randomization	No randomization was applied as the study did not involve large cohorts for treatment and control groups.
Blinding	Investigators were not blinded to group allocation during either data collection or analysis. Given the nature of the data and the analytical methods used, blinding was not deemed relevant to the integrity or objectivity of the study results.

Reporting for specific materials, systems and methods

We require information from authors about some types of materials, experimental systems and methods used in many studies. Here, indicate whether each material, system or method listed is relevant to your study. If you are not sure if a list item applies to your research, read the appropriate section before selecting a response.

Materials & experimental systems

n/a	Involved in the study
☒	☐ Antibodies
☐	☒ Eukaryotic cell lines
☒	☐ Palaeontology and archaeology
☒	☐ Animals and other organisms
☒	☐ Clinical data
☒	☐ Dual use research of concern

Methods

n/a	Involved in the study
☒	☐ ChIP-seq
☒	☐ Flow cytometry
☒	☐ MRI-based neuroimaging

Eukaryotic cell lines

Policy information about cell lines and Sex and Gender in Research

Cell line source(s)	HCC1143, HCC1806, HCC1937, HCC38, and MDAMB468 (cat#TCP-1001) cells were purchased from the American Type Culture Collection (https://www.atcc.org/products/tcp-1001).<br>BT549, CAL51, MDAMB231, MDAMB436 and SUM149PT cells were provided by the Sorger lab (Harvard Medical School, Ludwig Cancer Center, Boston, USA).<br>MCF10A and MCF7 cells were gifted by the Brugge lab (Harvard Medical School, Ludwig Cancer Center, Boston, USA).<br>GIMEN and SH-SY5Y cells were provided by the Schulte lab (Universitätsklinikum Tübingen, Clinic for Pediatrics and Adolescent Medicine, Thübingen, Germany)<br>U-2 OS reporter cell lines (wild-type, Cry1-sKO, Cry2-sKO, Cry1/Cry2-dKO were provided by the Kramer lab (Charité, Institute for Medical Immunology, Berlin, Germany).
Authentication	In this study we use a panel of ATCC-certified cell lines: HCC1143, HCC1806, HCC1937, HCC38 and MDAMB468. All cell lines were monitored for morphology, growth characteristics and health, mostly by long-term live-cell imaging, confirming that cell line-specific features did not vary throughout the study.
Mycoplasma contamination	Cell lines were tested for mycoplasma contamination and showed no infection, nor any signs of health decline.
Commonly misidentified lines<br>(See ICLAC register)	No commonly misidentified cell line was used in this study.
